# Association of *NFKB1* gene rs28362491 mutation with the occurrence of major adverse cardiovascular events

**DOI:** 10.1186/s12872-022-02755-x

**Published:** 2022-07-13

**Authors:** Jun-Yi Luo, Fen Liu, Tong Zhang, Ting Tian, Fan Luo, Xiao-Mei Li, Yi-Ning Yang

**Affiliations:** 1 Department of Cardiology, The First Affiliated Hospital of Xinjiang Medical University, 137 Liyushan South Road, Urumqi, 830054 Xinjiang China; 2 Xinjiang Key Laboratory of Cardiovascular Disease Research, Urumqi, Xinjiang China; 3 People’s Hospital of Xinjiang Uygur Autonomous Region, 91 Tianchi Road, Urumqi, 830054 Xinjiang China

**Keywords:** *NFKB1* gene, Major adverse cardiac event, Susceptibility

## Abstract

**Background:**

Several studies have reported that *NFKB1* gene rs28362491 polymorphism was associated with susceptibility to coronary heart disease in populations of different genetic backgrounds. To date, there have been no studies on the association between *NFKB1* gene rs28362491 polymorphism and the occurrence of major adverse cardiac and cerebrovascular event (MACCE). The present study was to explore the relationship between *NFKB1* gene rs28362491 polymorphism and MACCEs to investigate whether identifying *NFKB1* gene polymorphism is beneficial to evaluating MACCE risks and patients’ prognoses.

**Methods:**

We recruited 257 high-risk of cardiovascular disease patients with chest pain or precordial discomfort. The SNPscan™ were used to analyze the *NFKB1* gene rs28362491 polymorphism. All patients were followed up in the clinic or by telephone interview for MACCEs.

**Results:**

During the followed-up time (mean: 30.1 months) 49 patients had MACCEs (19.1%). Patients with the different genotypes of *NFKB1* rs28362491 had different incidence rate of MACCE. The incidence of MACCE in patients carried II, ID and DD genotype was 16.5%, 15.9%, 32.6%, respectively. Log-rank analysis showed that the survival rate in patients with *NFKB1* rs28362491 DD genotype was much lower than that in II or ID genotype carriers (*P* = 0.034). After excluding the influence of traditional risk factors of MACCEs, Cox regression showed that the DD genotype carriers had 2.294-fold relative risk of MACCEs comparing with patients carried II or ID genotype.

**Conclusion:**

The *NFKB1* gene rs28362491 mutant was an independent predictor of worse long-term prognosis for MACCEs. Therefore, identifying *NFKB1* gene rs28362491 mutant may be used as a good way for guiding the standardized management of patients with high-risk of cardiovascular diseases.

## Introduction

Cardiovascular diseases, especially atherosclerosis, have posed a serious risk to human health in China and worldwide. There are about 290 million people suffering from cardiovascular diseases in China, where the number of deaths due to these diseases accounts for approximately 41% of the total number of deaths, with cardiovascular diseases being the first cause of death within Chinese population [1]. Major adverse cardiovascular events (MACCEs) include all-cause death, death from cardiovascular diseases, stroke, angina pectoris, heart failure, need for target vessel revascularization due to coronary artery stenosis, and malignant arrhythmia, which are crucial indicators to evaluate the prognosis of cardiovascular diseases [2]. Reduction of the occurrence of MACCEs in populations, especially the population at high risk of cardiovascular diseases, is an important measure to improve the prognosis of cardiovascular diseases, and also, an important approach to reduce the economic burden on patients and the society. Therefore, finding molecular biomarkers associated with the occurrence of MACCEs in cardiovascular diseases and implementing effective clinical interventions to treat them are of great practical importance.

*NFKB1* gene, which is associated with metabolic inflammation, locates on human chromosome 4q24 and comprises 24 exons and 23 introns, encoding two key subunits of the NF-κB signaling pathway, p100 and p105, the latter of which can be enzymatically converted to a functional p50 subunit. There is an ATTG insertion/deletion polymorphism (rs28362491) at the -94 position in the promoter region of *NFKB1* gene, and this mutation is significantly associated with the susceptibility to various inflammatory diseases, such as Hashimoto's thyroiditis [3], ulcerative colitis [4], multiple sclerosis [5], left ventricular insufficiency [6], and dilated cardiomyopathy. Karban et al. [7] reported that the expression level of p50 protein in colon cancer cells was significantly reduced after deleting rs28362491. Park et al. reported that the deletion of rs28362491 led to a significant decrease in the transcriptional function of p50 in endothelial cells, and an increase in endothelial cell injury caused by blood flow shear force [8]. Therefore, rs28362491 mutation of *NFKB1* gene is considered as a functional polymorphism.

Several studies have reported that rs28362491 mutation is associated with susceptibility to coronary heart disease in populations of different genetic backgrounds [9–11]. To date, there have been no studies on the association between *NFKB1* gene polymorphisms and the occurrence of MACCEs. In the present study, we aimed to identify the genotype of *NFKB1* gene rs28362491 mutation in high-risk patients who visited our hospital for chest pain and tightness and were diagnosed with developed coronary artery stenosis but not yet coronary artery disease using coronary angiography, and also to follow up the patients for MACCEs to explore the association between the *NFKB1* gene mutations and the occurrence of MACCEs, and clarify whether *NFKB1* genotyping is beneficial for identifying the occurrence of MACCEs and improving the prognosis of patients with cardiovascular diseases.

## Subjects and methods

### Subjects

All subjects in this study were unrelated individuals who were long-term residents of the Xinjiang region, China, and they were admitted to the Heart Center of the First Affiliated Hospital of the Xinjiang Medical University with symptoms of chest tightness or precordial discomfort during 2015–2018. Each subject signed an informed consent before participating in this study. All subjects underwent coronary angiography after admission, and the diameter of coronary artery lumen was calculated as a percentage of the diameter of normal coronary artery lumen. Patients with ≥ 50% reduction in the diameter of one or more coronary artery lumens were excluded. The included patients were subjected to electrocardiogram analysis and cardiac enzyme (cardiac troponin I/T, cardiac enzyme spectrum) tests to exclude the patients with acute myocardial infarction according to the relevant diagnostic criteria of the American College of Cardiology/American Heart Association and the European Society of Cardiology [12, 13]. Additionally, those with incomplete data and complicated with one or more than one disease, such as secondary hypertension, rheumatic heart disease, congenital heart disease, heart failure, systemic immune system diseases, and multiple organ failure were excluded.

### Peripheral blood DNA extraction

A total of 5 mL of fasting peripheral venous blood was drawn from the subjects into ethylenediaminetetraacetic acid (EDTA)-containing blood collection tubes, and centrifuged at 5000 rpm for 5 min at 4 °C. Plasma and blood cells were separated and stored in a − 80 °C refrigerator until further use. Plasma was subjected to biochemical indicator testing and blood cells were subjected to genomic DNA extraction using a whole blood genome extraction kit (Tiangen Biotech, China).

### Identification of rs28362491 polymorphism in *NFKB1* gene

The genotypes of rs28362491 were identified using SNPscan™, a multiple SNP typing technology of Shanghai Tianhao Biotechnology Co., Ltd. This technology involves the following steps: (1) using allele-specific ligase detection reaction to identify the allele at a specific locus; (2) introducing non-specific sequences of different lengths via ligase ligation to obtain ligation products of different lengths of the corresponding locus; (3) using fluorescently labeled universal primers to PCR-amplify the ligation products and separate the amplified products using fluorescent capillary electrophoresis; and (4) analyzing the electrophoretogram to identify the genotype at the corresponding locus. The sequences of amplification and universal primers are provided in Table 1. The electrophoresis results revealed that the genotypes of rs28362491 were wild-type homozygous insertion (II genotype), variant homozygous deletion (DD genotype), and heterozygous (ID genotype).

**Table 1:** The sequences of amplification and universal primers of rs28362491

Primers	Sequences
Forward primer	5′-GCCTGCGTTCCCCGACCACTG-3′
Reverse primer	5′-TGCTGCCTGCGTTCCCCGTCC-3′
Universal primer	5′-ATTGGGCCCGGCAGGCGCTT-3′

### MACCEs follow-up

In this study, all subjects were monitored through follow-up at 6-month intervals through telephone or clinic visit, and the follow-up endpoint was the occurrence of one or more of the following MACCEs: all-cause death, death from cardiovascular diseases, stroke, angina pectoris, heart failure, need for target vessel revascularization due to coronary artery stenosis, and malignant arrhythmia.

### Statistical analysis

Statistical analysis was performed using SPSS v.22.0. The measurement data were expressed as mean ± standard deviation ($\overline{X }$±SD), and the comparison of means between the groups was performed using the independent samples t-test. The count data were expressed as the number of cases (%), and comparison of frequencies between the groups was performed using the χ^2^ test. Differences in the occurrence of MACCEs among patients with different genotypes of *NFKB1* gene rs28362491 mutation were evaluated using the log-rank test. The influencing factors associated with the occurrence of MACCEs were evaluated using the Cox regression analysis. The difference was considered statistically significant when the two-sided *P* < 0.05.

## Results

### General clinical characteristics

A total of 257 patients at high risk of cardiovascular diseases were included in this study, of whom 49 (19.1%) experienced at least one MACCE. The age, plasma low-density lipoprotein cholesterol (LDL-C) levels, and proportion of patients with hypertension were significantly higher (all *P* < 0.05) in the MACCE group than in the non-MACCE group (Table 2).

**Table 2:** Demographics, clinical baseline characteristics

	No-MACCE (n = 208)	MACCE (n = 49)	*P* value
Age, mean (year)	58.1 ± 12.8	62.3 ± 12.6	0.039
Male (n, %)	182 (87.5)	38 (77.6)	0.074
BMI (kg/m^2^)	25.1 ± 3.1	25.2 ± 3.5	0.822
SBP (mmHg)	121.7 ± 19.5	127.3 ± 24.5	0.085
DBP (mmHg)	76.5 ± 13.2	75.7 ± 15.2	0.714
Diabetes (n, %)	45 (21.6)	15 (25.0)	0.181
Hypertension (n, %)	84 (40.4)	29 (59.2)	0.017
TG (mmol/L)	1.8 ± 1.2	1.6 ± 0.8	0.253
TC (mmol/L)	3.8 ± 0.9	4.0 ± 0.8	0.151
LDL-C (mmol/L)	2.4 ± 0.7	2.7 ± 0.7	0.016
HDL-C (mmol/L)	0.9 ± 0.2	0.9 ± 0.2	0.121

Continuous data were presented as mean ± SD. Categorical data were presented as the number (percentage)

MACCE, major adverse cardiac and cerebrovascular event; BMI, body mass index; SBP, systolic blood pressure; DBP, diastolic blood pressure; TG, triglycerides; TC, total cholesterol; LDL-C, low density lipoprotein-cholesterol; HDL-C high density lipoprotein-cholesterol

### Occurrence of MACCEs in patients with different genotypes of rs28362491 in *NFKB1* gene

**Fig. 1:**
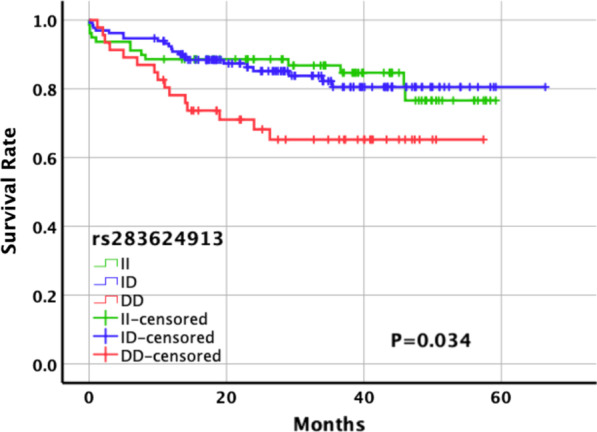
Kaplan–Meier curves for MACCEs according to different genotypes of *NFKB1* gene rs28362491

In the present study, the mean follow-up duration was 29.5 (17.1, 43.75) months. Among the subjects with II, ID, and DD genotypes of *NFKB1* gene rs28362491 mutation, 16.5%, 15.9%, and 32.6% developed MACCEs, respectively, and the distribution among the three genotypes in the two groups was statistically different (*P* = 0.036) (Table 3). The log-rank test revealed that the survival rate was significantly lower in the patients with DD genotype of *NFKB1* gene rs28362491 than in those with II or ID genotypes (*P* = 0.034) (Fig. 1).

**Table 3:** MACCEs among high-risk of cardiovascular disease patients carrying different genotypes of *NFKB1* gene rs28362491

	No-MACCE (n = 208)	MACCE (n = 49)	*P* value
rs28362491 genotypes			
II	66(83.5)	13(16.5)	
ID	111(84.1)	21(15.9)	0.036
DD	31(67.4)	15(32.6)	

MACCE, major adverse cardiac and cerebrovascular event

### Risk factors of MACCEs

In the present study, we investigated the following risk factors of MACCEs, including the age, sex, hypertension, diabetes, total cholesterol level, triglyceride level, and LDL-C level, as well as the *NFKB1* gene with a difference in genotype distribution between the MACCE and non-MACCE groups. The cox regression analysis revealed that after adjusting the effects of age, sex, disease status, and lipid level on the two groups, the DD genotype of *NFKB1* gene rs28362491 was an independent risk factor associated with the occurrence of MACCEs, and the hazard ratio (HR) of MACCEs for DD genotype was 2.294 (95% CI 1.227–4.288, *P* = 0.009) times than that for II or ID genotypes (Table 4).

**Table 4:** Predictors of MACCEs by Cox regression

Risk factors	HR	95%CI	*P* value
rs28692491 genotypes			
DD versus II + ID	2.294	1.227–4.288	0.009
Male	1.495	0.708–3.157	0.291
Age	1.016	0.993–1.040	0.165
Hypertension	1.598	0.875–2.919	0.127
DM	1.471	0.781–2.768	0.232
TG	0.856	0.610–1.203	0.371
LDL-C	2.385	0.891–6.388	0.084
TC	0.664	0.281–1.569	0.351

HR, Hazard ratio; CI, confidence interval; DM, diabetes mellitus; TG, triglycerides; LDL-C, low density lipoprotein cholesterol; TC, total cholesterol

## Discussion

A total of 257 patients at high risk of cardiovascular disease were included in this study, with a median follow-up duration of 29.5 months, and a total of 49 cases were with MACCEs. There were significant differences in the frequency of the three genotypes of *NFKB1* gene rs28362491 between the MACCE and non-MACCE groups, and the survival rate was significantly lower in the patients with DD genotype than in those with II or ID genotypes. The Cox survival regression analysis revealed that after adjusting for the effects of the traditional risk factors of MACCEs, the HR of MACCEs for DD genotype was 2.294 times higher than that for II or ID genotypes, suggesting a significant association between the rs28362491 polymorphism in the promoter region of *NFKB1* gene and occurrence of MACCEs.

NF-κB, an important transcription factor, is widely found in a variety of cells and exhibits a multidirectional regulatory function for gene transcription, thereby regulating the gene transcription of a variety of cytokines and chemokines [14]. The activated NF-κB can be detected in atherosclerotic (AS) plaques, and it is known to promote the expression of downstream target cytokines and pro-inflammatory factors, such as interleukin-6 and -1β (IL-6, IL-1β), tumor necrosis factor-α and P-selectin, monocyte chemotactic protein-1, intercellular adhesion molecules, and vascular cell adhesion molecules at the gene transcription level, which in turn leads to the formation of AS plaques [15, 16]. Additionally, NF-κB is also involved in regulating the expression of smooth muscle cell proliferation-related cytokines to promote the formation of AS plaques. In a number of studies, including our previous studies, it has been shown that gene polymorphism at the -94 position of the *NFKB1* gene promoter region is associated with the susceptibility to coronary heart diseases in populations of different genetic backgrounds. In our previous studies, we have also shown that the deletion mutations at the position reduce p50 expression and its transcriptional function of the NF-κB signaling pathway, down- and up-regulate the mRNA expression level of endothelial-type nitric oxide synthase, and inflammatory cytokine IL-6 genes, respectively, leading to endothelial cell dysfunction, apoptosis, and injury, thereby increasing the susceptibility to coronary heart disease [17]. These findings indicate an important role of *NFKB1* gene mutation in the pathogenesis of coronary heart diseases.

In most studies, the role of genes in the pathogenesis of cardiovascular diseases has been investigated, and research has clearly shown that cardiovascular diseases are a type of genetic diseases associated with multiple genes, with genetic factors accounting for up to 40% of the incidence of coronary heart diseases [18]. Susceptibility genes are innate risk factors that individuals possess throughout their lives, and it has become extremely important to first screen the individuals at high risk of cardiovascular diseases using genetic testing before the occurrence of these diseases, and then to make appropriate lifestyle or pharmacological interventions, which is of great practical importance for the prevention of cardiovascular diseases. The results of the present study suggests that there are differences in the incidence of MACCEs among patients who are at high risk of cardiovascular diseases with different *NFKB1* genotypes, with the HR of MACCEs for DD genotype being 2.294 times higher than that for II or DD genotype, suggesting that *NFKB1* gene mutation are a high-risk factor for the prognosis of cardiovascular diseases. Studies on non-Chinese populations have shown that the risk scores that include genetic factors can improve the prediction of coronary heart disease, hypertension, and lipid levels relative to the risk prediction models that include only traditional risk factors, particularly for improving the prediction in the population with intermediate risk of cardiovascular diseases [19–21]. It was reported that the genetic risk scores were significantly associated with a linear increase in blood pressure, and the risks of hypertension and cardiovascular disease, and that the genetic risk scores helped to improve the discrimination between hypertension and cardiovascular disease, and improve the risk stratification for cardiovascular diseases [22]. Subsequently, study of genome-wide association analysis on the lipid level in 8,344 subjects found that the genes *LPL*, *TRIB1*, *APOA1-C3-A4-A5*, *LIPC*, *CETP*, and *LDLR* were correlated with the lipid level changes, and that the individual genetic variation and cumulative effects were independent risk factors associated with the increase in lipid levels and hyperlipidemia [23]. Moreover, researchers developed a genome-wide multi-gene scoring model for five common diseases, using which they identified more than threefold increase in the risk of coronary heart diseases in 8.0% of the population, and up to 19-fold increase in the risk of coronary heart diseases in individuals with rare single-gene mutations [24]. Khera et al. performed a prospective cohort study on individuals at high risk of cardiovascular diseases, finding that those with a high genetic risk had a 46% reduction in the risk of coronary events after implementing healthy lifestyle interventions, such as non-smoking, weight control, regular physical activity, and healthy diet, and that the risk of coronary events was significantly associated with coronary calcification [25]. In the present study, we also clearly demonstrated that the patients at high risk of cardiovascular diseases with *NFKB1* gene DD mutant genotype have a significantly higher risk of MACCEs than those without mutant genotypes. Therefore, it is necessary to identify *NFKB1* genotypes in the population at high risk of cardiovascular diseases, and for those with DD genotype aggressive clinical pharmacological and healthy lifestyle interventions should be implemented to reduce the occurrence of MACCEs.

Conclusively, in the present study, we confirmed that DD genotype of *NFKB1* gene is an independent risk factor associated with MACCEs, and detection of rs28362491 polymorphism in *NFKB1* gene is beneficial for identifying patients who are at high risk of cardiovascular diseases, for whom the occurrence of MACCEs may be reduced through effective interventions. Additionally, genomics-based personalized therapy must be considered as the future direction of precision medicine research, and the present findings provide potential intervention targets for the prognosis of patients who are at high risk of cardiovascular diseases.

## Data Availability

All of the data were presented in the main paper. The data that support the findings of this study was submitted to the dbSNP database in NCBI (ID: SUB11037636).
